# Bouts, Pauses, and Units of Operant Performance: A Primer

**DOI:** 10.1007/s40614-024-00419-z

**Published:** 2024-08-30

**Authors:** John Michael Falligant, Louis P. Hagopian, M. Christopher Newland

**Affiliations:** 1 Kennedy Krieger Institute, Baltimore, MD USA; 2 Johns Hopkins University School of Medicine, Baltimore, MD USA; 3 Auburn University, Auburn, AL USA

**Keywords:** Behavioral neuroscience, Bouts, Log survivor, Interresponse time, Temporal dynamics

## Abstract

Operant behavior typically occurs in bouts and pauses. The microstructural analysis of bouts and pauses reveals important and separable information about the physical characteristics of the operant and the motivation behind it. An analysis of interresponse times (IRTs) often reveals a mixture of two exponential distributions. One corresponds to short IRTs within ongoing response bouts, reflecting motor properties of the operant, and the other corresponds to longer intervals between bouts, reflecting the motivation behind the response. Partitioning responses into bout initiations and within-bout responses via this two-mode framework reveals the mechanisms underlying behavior maintenance and change. This approach is used in the fields of neurotoxicology, behavioral pharmacology, and behavioral neuroscience to disentangle the contribution of motivational and motoric variables to the pattern of operant behavior. In this article, we present a primer aimed at providing essential concepts related to the analysis of response bouts and temporal dynamics of operant performance.

Response rate has long been a primary dependent variable in the experimental analysis of behavior (Skinner, 1938). The early writings of Skinner fixed patterns of response rate as the key to characterizing environmental determinants of behavior (Skinner, 1936/1981). The early primacy of cumulative records in behavioral psychology explicitly reflects the emphasis of response rate as a primary measure of behavior, especially how it changes in time at a molecular level (Ferster & Skinner, 1957; Kangas & Cassidy, 2011; Skinner, 1938). Response rate is fundamental to our understanding of behavior as choice (Herrnstein, 1961), behavioral momentum (Nevin, 1988), relapse and resurgence (Podlesnik & Shahan, 2010), and the theoretical system known as Mathematical Principles of Reinforcement (Killeen, 1994). Response rate is also a fundamental measure of behavior maintenance and change central to many areas of applied behavior analysis (Johnston & Pennypacker, 1993).

Response rate is typically determined by dividing the total number of responses (e.g., 20 lever presses) by the duration of the available time (e.g., 600 s) to yield an average response rate (e.g., 0.03 responses per s or 2 responses/min). Average response rate is the simple inverse of the average interresponse time (IRTs) that separates each occurrence of behavior. High-rate behavior necessarily comprises short IRTs, whereas low-rate behavior comprises long IRTs (Killeen et al., 2002). Although overall response rate can be a useful metric for analyzing behavior–environment interactions in many circumstances, it misses details about response dynamics and the microstructure of behavior contained in the cumulative record. In a sense, calculating response rate simply as the total number of responses per available duration of time implicitly assumes that all IRTs are more-or-less equivalent; that is, generated by a single process and described by a single probability distribution (Shull et al., 2001; Sibly et al., 1990). This discounts the potential relevance of ebbs and flows in the stream of behavior. As noted by Shull et al. (2001): “This method makes more sense if all instances of the response are functionally equivalent. But there are grounds for thinking that they might not be, at least not under some widely studied conditions” (p. 247).

One perspective holds that operant behavior is organized into periods of engagement in a target response alternating with periods of disengagement from the target response in which the organism engages in other activities (Shull et al., 2001). This view accords with decades of ethological work indicating that animal behavior in natural environments occurs in bouts and pauses. In this article, we present a primer to outline foundational knowledge and review essential concepts related to the analysis of response bouts and temporal dynamics of operant performance. By emphasizing core concepts, we aim to establish a base for further exploration and application of this perspective for applied and translational researchers. For researchers, we will illustrate how bout analyses separate motivational and motor determinants of a bout, which can be important to understanding both behavioral and neural mechanisms of behavior change. We will also illustrate which components of response bouts are responsible for higher-order behavioral effects, such as response transitions in a test of behavioral plasticity. For clinicians, we will show how an appreciation of the microstructure of behavior may yield insight about the determinants of socially significant behavior—insights that potentially indicate when or why certain interventions might be effective, as well as how other physical factors might be revealed in patterns of operant performance.

Bouts generally refer to periods of responding (e.g., drinking, preening, running, lever-pressing) separated by periods of disengagement in the response of interest in which the animal engages in alternative activities (e.g., foraging, grooming, eating). Barking is an example that most dog owners can readily identify with, because it generally occurs in bouts (e.g., Flint et al., 2013). Between bouts of barking, dogs engage in myriad alternative behaviors (e.g., licking, playing, eating, drinking, chewing, getting into the trash, sleeping). Bout characteristics of barks vary, depending on context. For example, following a disturbance in the environment (e.g., doorbell rings, mail truck arrives), bouts of barking occur for an extended duration of time, and intervals between barks are shorter relative to contexts without disturbances when dogs are playing or isolated from their owners.^1^ In this example, bouts are separated by intervals that are longer than the inter-bark interval seen within a bout (Machlis, 1977; Sibly et al., 1990). In other words, *bouts* are states of behavioral engagement consisting of some high-rate response with a quantifiable duration (Hoffman & Newland, 2016). The bout pattern for the barking example requires several elements to describe, including (1) the *bout-initiation rate* (i.e., how frequently the dog initiates barking episodes); (2) *bout length* (i.e., the number of barks constituting a bout); and (3) the *within-bout response rate* (the rate at which the dog barks once a bout has started). These components of response bouts can be quantified using nonlinear regression modeling, with different coefficients representing the three bout parameters. How one goes about estimating these parameters will be developed later in this article. First, we offer a conceptual introduction to this bout-analytic framework.

Premack (1965) was one among many to note that a two-mode response pattern characterizes many activities observed in laboratory animals (Baum, 2002; Choleris et al., 2001; Mechner, 1992; Nevin & Baum, 1980; Shull et al., 2001; Shull, 2011; see also Catania, 2013; Gallistel, 1981; and Timberlake, 1983, for some related perspectives). Results from a long line of research indicate that reinforced responding is composed of periods of engagement in the reinforced activity (sometimes called “visits”; Shull, 2011) alternating with periods of disengagement from the target activity and engagement in some alternative activity (Herrnstein, 1970; Shull, 2011; Shull et al., 2004). Each target response can be classified as either a bout-initiation or a response occurring within an ongoing bout (Kulubekova & McDowell, 2008). According to this perspective, certain types of performance may be analogous to a constant-speed motor or drill that can only be in one of two states: on or off. Once the drill is on (i.e., bout initiation), it rotates at a constant speed (i.e., within-bout response rate) for some amount of time or number of revolutions (i.e., bout length). The between-bout pauses, the inverse of bout-initiation rate, reflect the duration of time the drill is off.

Steller and Hill (1952) noted that rats experiencing varying levels of water deprivation displayed this two-mode pattern: the rate of initiating drinking bouts was a negatively accelerated function of how long the rats had been deprived of water before the test. Deprivation level primarily affected the duration of pauses between bouts of drinking and the duration of those bouts, but it did not affect the rate of drinking within bouts (cf., Cone, 1974). Like a constant-speed drill, once a bout began it occurred in a stereotyped fashion driven by the physical properties of the act itself. As Steller and Hill remarked, “Under all conditions, the rat drinks at a constant rate or it does not drink at all” (p. 102, as cited in Shull, 2011).

Reinforcer magnitude effects on fixed-ratio (FR) schedules are characterized by similar patterns (see Shull, 2011). For example, discriminative stimuli signaling large-magnitude reinforcers (10-s grain access) are associated with higher overall rates of key pecking relative to discriminative stimuli signaling lower-magnitude reinforcers (5-s grain access; Morse, 1966). It is interesting that, similar to the water deprivation example from Steller and Hill, the difference in overall response rate is attributable to differences in the duration of pauses between bouts and not different rates of responding within bouts (Shull, 2011). In another example involving punishment, Azrin (1959) demonstrated that the addition of a punishment contingency (FR 1 brief electric shock) to a FR 25 food reinforcement schedule decreased pigeons’ key pecking due to lengthening the duration of the pauses in-between bouts and not by way of within-bout responses rates (Shull, 2011). As summarized by Azrin (1959),

It is seen that punishment affects fixed-ratio performance by selectively increasing the postreinforcement pause. The greater the intensity of punishment, the longer the pausing. Once responding begins, the local rate remains virtually unchanged regardless of the intensity of punishment. Any reductions in the total number of responses by punishment, therefore, is attributable to an increase in the pauses, not to any decrease in the local rate of responding. (pp. 303–304)

Similar patterns were also reported in early studies with humans, rats, cats, pigeons, and a rhesus monkey (Azrin, 1958; Ferster & Skinner, 1957; Sidman & Stebbins, 1954). These studies strongly suggest that the pausing between bouts is linked to motivational states.

## Impact of Motoric and Motivational Variables on Bout Structure

The univariate approach to calculating response rate (responses divided by available time) contrasts with work by Shull et al. (2001) suggesting that overall response rate is a composite measure of performance. That is, response rate may appear to be a single quantity, but it actually comprises two, and maybe more, dissociable elements. An extensive body of research has demonstrated that experimental manipulations of motoric, schedule, and motivational variables differentially affect the temporal dynamics of responding.

Some definitions are warranted here. Motor determinants are those variables that affect the biophysical properties of movement and action. The term “biophysical” is used to draw attention to both the structure on which movement occurs (a surface being walked on, a lever being pressed, etc.) and as the physiological or anatomical constraints on movement (muscle dynamics, bone length, tendon structure, etc.). Changes to motoric variables (Brackney et al., 2011; Jiménez et al., 2017; Johnson et al., 2011) or physical impairment caused by exposure to an environmental contaminant (Hoffman & Newland, 2016; Shen et al., 2016a, 2016b), selectively affect within-bout response rates once a bout has been initiated. Some reinforcement schedule manipulations, especially those that select high- or low-rate responding, can also affect within-bout rate, as discussed below.

Motivational determinants are those factors that influence which response will occur in a context of all available responses. Establishing operations—such as caloric or fluid restriction, removing preferred attention, or the availability of alternative reinforcers—can, in this view, affect whether a bout of behavior will commence (Herrnstein, 1970; see also Smith et al., 2014).

One component of response rate is determined by motivational variables and manifests as the bout-initiation rate, whereas the other is controlled by variables affecting the motor components of behavior and manifests as the within-bout response rate (Shull et al., 2001). This makes sense because the bout-initiation rate is a measure of how frequently an organism initiates responding, making the transition from doing “something else” to engaging in the target behavior under study. Thus, changes to motivational variables affect bout-initiation rates, or how often responding is initiated (Johnson et al., 2009; Podlesnik et al., 2006; Reed, 2011; Reed et al., 2018; Shull, 2004, 2011; Shull & Grimes, 2003; Shull et al., 2001, 2002, 2004; Smith et al., 2014). Operations that act on motivational variables—including reinforcement rate, quality, deprivation, and alternative reinforcer availability—affect the bout-initiation rate (Johnson et al., 2009; Shull, 2004, 2011). Bout-initiation rate reflects the availability of external reinforcers, such food for lever-pressing (Shull et al., 2001). It also reveals the value of presumably endogenous reinforcers seen with automatically reinforced behavior, like wheel-running in rodents (Johnson et al., 2009, 2011; Shen et al., 2016a) and possibly self-injurious behavior (Falligant 2024). In contrast, the within-bout response rate is controlled by motoric or effort-related factors, such as pharmacologic agents (Johnson et al., 2011), exposure to debilitating environmental contaminants (Hoffman & Newland, 2016), manipulations that change the physical effort associated with the response or the form of the response unit (e.g., Brackney et al., 2011), and genetic factors (Johnson et al., 2009).

## Experimental Analyses of Bout Structure

Increases/decreases in response rate can arise via patterns of change in any or all these constituent measures that are sensitive to different variables. This view of behavior assumes that (1) bout components are correlated with response rate and (2) they are not correlated with each other (e.g., Falligant 2024; Johnson et al., 2011). Johnson et al. (2009) examined this question by determining bout structures in two mouse strains, using running wheels and nose poking jointly or separately, and after reversing the photoperiod and session prefeeding. One question in this study was whether the three bout measures (i.e., bout-initiation rate, within-bout response rate, bout length) were correlated with overall response rate and to what experimental operations they were sensitive. In this study, nose poking in mice was maintained under a random interval schedule that reinforced criterion IRTs, as determined by a percentile schedule, on average every 60 s (RI 60″). To identify criterion IRTs, a percentile schedule compared a current IRT with the previous 10: if the IRT was shorter than half of the previous 10, it was eligible for reinforcement under the RI schedule. All criterion IRTs produced a brief food-paired tone.^2^ Although complicated to describe, behavior under this percentile schedule arrangement is acquired quickly and maintained robustly. A significant advantage is that overall reinforcer rate is held constant against a range of response patterns because the criterion adjusts according to an animal’s most recent performance, so changes in reinforcement rate do not complicate conclusions drawn from the results. Bout-initiation rates of nose poking were shortened by pre-feeding, the absence of a running wheel (alternative reinforcers), and the photoperiod (which determines the time of day that food is most reinforcing). These are motivational variables. Within-bout rate was influenced by mouse strain, which is a genetic variable that had other complex effects. Bout length of nose poking was affected by the presence of a running wheel: bout lengths were longer when the wheel was absent. The second question in this study was whether the bout components offered independent measures of behavior. It is striking that the bout parameters were not correlated with each other. Thus, these composite measures offered independent assessments of important determinants of behavior, suggesting that they are indeed orthogonal.

To examine the variables underlying behavior maintenance and change, Brackney et al. (2011) applied a response-bout analysis to data from rats’ lever pressing maintained by sucrose on variable-interval (VI) schedules. The rats were free-fed, but the sucrose reinforcer was sufficient for maintaining behavior. A variety of motoric, motivational, and schedule variables were manipulated. The effort-related manipulation consisted of changes in the physical requirement for pressing the lever. A “low workload” lever was positioned 21 mm above the floor with a force activation requirement of 0.05 N, and a “high workload” lever was positioned 165 mm above the floor with a force activation requirement of 0.78 N. The deprivation-related manipulation consisted of removing chow from the rats’ home cage for 24 h prior to the experimental session. The schedule-related manipulations entailed a range of VI schedules (e.g., VI 5 s to VI 120 s) and a tandem variable-time (VT) 120-s FR 5 schedule similar to that used by Shull et al. (2001). Under the tandem schedule,^3^ five responses had to be executed after the VT component timed out before food was delivered, so there was both a time and response requirement. Ratio schedules reinforce short IRTs (Rasmussen et al., 2022; Shull et al., 2001; Zeiler, 1977), so the tandem VT FR schedule would be expected to increase the within-bout rate.

Brackney et al. (2011) introduced a new variable called the refractory period. This can be viewed as the shortest IRT that is possible given the biophysical constraints present. For example, if the operant is the completion of an arithmetic problem, then no IRT (the time between the onset of one response and the onset of a second response) can be shorter than the shortest time to complete a problem. This would be longer than, say, the time required for a mouse to nose poke or lever press. But the idea also applies to these short response durations. In a sense, Shull et al. (2001) introduced a form of refractory period before conducting their bout analyses indirectly by analyzing only IRTs longer than one second because doing so “seemed to work well for our data sets” (p. 252). Brackney et al. found their putative effort-related manipulation—increasing the physical workload and elevating the lever—lengthened the refractory period and decreased bout-initiation rates, but had no effect on bout length or—and this is interesting—within-bout rate. This suggests that the refractory period is a good indicator of the biophysical interface between behavior and the physical properties of what behavior acts on. This response effort-related manipulation appeared to increase the pausing before a new response bout was initiated, and therefore might be construed as acting on motivation, but had no effect on within-bout rate. Shull et al. (2001) speculated that within-bout rate reflected motor properties of behavior, which the work we describe below involving drugs and environmental contaminants also suggests. Nonetheless, the absence of an effect on within-bout responses indicates that there is much to learn about how physical challenges to a behavior affect its bout structure. Regarding the other bout parameters, Brackney et al. found that caloric restriction selectively increased bout-initiation rates. Said another way, caloric restriction decreased the duration of pausing in-between bouts, which is consistent with many other studies reviewed herein. The introduction of a small FR requirement at the end of an interval schedule in the tandem schedule condition yielded higher overall response rates relative to interval schedules without the FR requirement; it did so by increasing bout length and within-bout response rate, as Shull et al. (2001) had reported.

## Motor and Motivational Contributions to Behavior

Unlike Brackney et al.’s (2011) finding that effort-related manipulations have effects similar to motivational variables, Johnson et al. (2009) reported that motor and motivational variables are orthogonal to one another. The reasons for the discrepant conclusions are not clear at present, but an important factor might be the way effort was altered in Brackney et al. (2011). A compelling demonstration of the separation of motor and motivational influences comes from studies involving exposure to an environmental contaminant, methylmercury, which severely impairs sensorimotor function. In these studies, C57Bl/6 mice were exposed chronically to low levels of methylmercury (MeHg) to model human exposure to this contaminant. MeHg is well-known to cause severe sensorimotor deficits after exposure to high doses (Day et al., 2005; Dietrich et al., 2005; Harada, 1995; Heath et al., 2010; Yorifuji et al., 2008). The term *sensorimotor* is used deliberately because MeHg-induced deficits could arise from sensory numbing, direct damage to motor systems, or both. These studies focused on (1) characterizing the behavioral deficits caused by low-level exposure to MeHg, and (2) testing certain theories about the neural mechanisms by which MeHg is neurotoxic by seeking to successfully protect mice from its harmful effects. Another specific interest was in whether both neurotoxicity and neuroprotection differentially affected sensorimotor and motivational endpoints. To address this last interest, bout analyses of different behaviors (e.g., wheel running, lever pressing) were conducted following MeHg exposure. Commensurate with a long-held dictum of behavioral pharmacology and toxicology, it was thought that a close examination of the controlling variables after chemical exposure can reveal important information about its structure and function (Branch, 1984).

In separate studies, the bout structure of wheel running and lever pressing was examined. Wheel running required no reinforcement contingencies. Lever pressing was maintained by a similar arrangement described above in which a percentile schedule determined the criterion, and short IRTs were reinforced under a random interval schedule. This was important because reinforcer rates were held constant even in the face of large decreases in lever-pressing rates due to the adjusting property of the percentile schedule. Response rates decreased during exposure due to reductions in within-bout rate for both wheel running (Hoffman & Newland, 2016; Shen et al., 2016a) and lever pressing (Shen et al., 2016b). The decrease in within-bout rate was the earliest and most sensitive indicator of neurotoxicity, because only later were overt motor deficits such as ataxia apparent. It is interesting that the bout-initiation rate was unaffected, even in the face of clearly visible sensorimotor deficits. Thus, wheel-deprived or caloric-restricted mice were still motivated to run or lever press, even when their ability to do so was impaired by neurological deficits. So, it is possible to separate these two determinants of the microstructure of behavior. These findings also suggest that operations affecting motoric factors can sometimes, but not necessarily, also change motivation: both factors can be assayed and isolated within this two-mode bout-analytic framework. The dissociable contributions of within-bout rate and bout-initiation rate to operant performance again highlight the fact that overall response rate is an inadequate index of motivation, and the appropriate unit of operant performance may be found in behavior’s microstructure.

Partitioning responses into bout initiations and within-bout responses via this framework facilitates analysis of the mechanisms underlying behavior maintenance or change. For example, suppose that two drugs (Drug A and Drug B) are both found to reduce food-maintained lever pressing. Examination of the response-bout characteristics reveals that Drug A decreases the number of bout initiations whereas Drug B decreases the within-bout response rate. The conclusion here is that Drug A reduces response rates by affecting motivational factors (a change in the reinforcing value of the consequence maintaining the operant), whereas Drug B reduces response rate by decreasing the speed or efficiency with which the organism can produce the target response while leaving the underlying motivation unchanged. In this example, a response-bout analysis can inform us as to the mechanism responsible for the drug-induced suppression of response rates, as described above with MeHg-impaired mice—a separation of motivational and motor determinants of behavioral deficits that had previously been elusive.

Although this conceptual and analytic approach has yet to be applied in the clinical realm, it may have utility. Imagine that a client with socially maintained challenging behavior earns tokens on a VI 4-min token production schedule for completion of simple vocational tasks (e.g., folding towels, sorting folders). These tokens are exchanged throughout the day to access preferred activities (e.g., playing video games). To increase the client’s rate of vocational task completion, one could make several manipulations: (1) increase the deprivation level of the preferred activities (e.g., changing video game access from an open economy to a closed economy); (2) increase the rate of the reinforcer delivery by changing the VI schedule from a VI 4 min to a VI 2 min; (3) increase the magnitude or quality of the backup reinforcer (e.g., allow longer play or access to better video games); (4) reduce the availability of alternative or substitutable reinforcers (e.g., limiting access to televisions, music, and computer time); or (5) add a small FR schedule requirement (e.g., FR 2) to the end of the VI schedule. In this tandem VI FR arrangement, the client would have to, for example, sort two additional folders after the VI response requirement had been met in order to produce the exchange period. According to the Shull et al. (2001) analysis, all these manipulations should increase the overall rate of vocational tasks for a given observational period. However, the first four manipulations can be grouped together as operating on motivational variables that change response rate by altering the relative reinforcement associated with the target response. The last manipulation (i.e., the tandem VI 4 FR 2 schedule), however, does not operate by inducing a motivational change affecting the relative value of the reinforcer. Rather, it works by affecting *the form or microstructure of the behavioral unit* rather than the individual’s *propensity to initiate that behavioral unit* (Shull et al., 2001). Although these manipulations will all increase the overall response rate, these different variables impart distinct temporal signatures on changes in bouts of target activity.

## Temporal Dynamics of Behavior Change

The interactions of operant behavior with environmental contingencies can be viewed as a dynamic system, in which the IRT is a fundamental unit of analysis akin to an atom in the study of physics (Marr, 1992). Analyzing steady state behavior has its own complexities, but transition states present an additional challenge because they are nonstationary. With steady state responding, entire sessions or even multiple sessions can be the source of IRTs. However, with transition states there is not a stable source of behavior because IRTs may fluctuate dynamically within an observational period. Two general approaches have been taken to address this issue. One is to examine different phases of a transition stage, such as sessions or components of a session (e.g., Palya et al., 1996). A second is to incorporate a coefficient for time in the session as a part of the model describing behavior. Thus, in addition to a term for each of the bout parameters—within-bout response rate, bout-initiation rate, bout length and, sometimes, refractory period—additional terms for time might be added (Brackney et al., 2017; Cheung et al., 2012).

In what may be the earliest attempt to examine the relative sensitivity of short and longer IRTs during transition states, Blough (1963) reported the differential sensitivity of short and long IRTs to changes in reinforcement contingencies. During extinction from a VI 4 min schedule, the short IRTs generated by pigeons remained relatively stable, but the long IRTs did not. Thus, the reduction in response rates arose from increases in the between-bout pauses and not from changes to within-bout responses. In other words, the IRTs forming the bouts changed only marginally but those forming the interbout intervals (i.e., the pause duration in-between bouts) showed large increases and primarily contributed to the decline in response rates during extinction. Subsequent work has shown that long IRTs, but not short IRTs, readily come under stimulus control (Blough, 1969) and short IRTs are relatively insensitive to schedule effects (Sewell & Kendall, 1965; White, 1985; see also, Davison, 2004), which could be pertinent to understanding why they are sensitive to behavior change.

To analyze bout structure during extinction, Shull et al. (2002) generated log survival plots from IRTs taken from sequential components of an extinction session using rats whose nose poking was reinforced by food pellets. The investigators were interested not only in how the microstructure changed during extinction but also in how background reinforcement rate, produced by noncontingent VT schedules, influenced bout components and how they aligned with behavioral momentum theory. This theory holds that extinction from a rich context is more resistant to change than extinction from a lean one (Nevin et al., 1983). The question here is whether this differential sensitivity is due to the bout-initiation rate or the bout length. As extinction transpired, the reduction in response rates was due to a decrease in bout-initiation rate; the longer IRTs became longer, just as Blough (1963) had reported. Likewise, the differential resistance to change seen in the rich and lean components was due largely to differences in the bout-initiation rate. The bout length was only modestly affected, at best, by extinction and the richness of the context. Extinction produced fewer bouts, but the length of those bouts changed very little. The key finding here is that behavioral momentum is affected mainly by the rate at which bouts occur and not bout length, an observation that would not have been possible by examining response rates alone.

Brackney et al. (2017) examined three behavior-reduction strategies in rats whose lever pressing was reinforced by sucrose pellets. They used a regression equation that included a term for time because the intervention was imposed in addition to terms for bout-initiation rate, within-bout rate, and bout length. This approach represents a quantification of Blough’s (1963) attempt to track structural changes continuously. Brackney et al. exploited the quantification available in their model by comparing the rate with which each parameter changed across interventions. The nature of changes in bout structure depended on which behavior reduction intervention was used and, in some details, the rat strain. Extinction reduced bout-initiation rate, similar to what has been reported by Shull et al. For one strain, extinction also reduced within-bout rate, and marginally reduced bout length. This points to the highly disruptive effect of extinction on response microstructure. Noncontingent reinforcement (NCR) shortened bout length, which, the authors suggest, could result from the adventitious reinforcement of behavior that competes with lever pressing, disrupting the relation between reinforcement and the target response. This article illustrates the value of incorporating time-based changes in the analysis of how decelerating interventions change behavior. It also illustrates again how reliance on response rates alone masks controlling variables.

In a very different application, a bout analysis was used to characterize the microstructure of behavior during the transition from high-rate to low-rate lever pressing among rats exposed during gestation to MeHg (Newland et al., 2013). The acquisition of low-rate behavior was viewed as a marker of response inhibition or, on the other hand, of motor impulsivity. Having shown in other studies that gestational methylmercury exposure imposed a behavioral rigidity to changes in spatial and visual discrimination in adults, Newland et al. were interested in whether it also imposed such rigidity to patterns of motor performance. This maps onto distinctions between cognitive and motor rigidity (Dalley et al., 2004). Lever pressing by rats was established under a percentile schedule in which IRTs were eligible for reinforcement only if they were shorter than the shortest 25% of the previous 10 IRTs. Eligible IRTs were reinforced unpredictably after an average of every 30 s (RI 30″ schedule). This established a high response rate (short IRTs) with a criterion that adjusted according to the animal’s recent performance, an important property when studying behavioral effects during impairment by drug or neurotoxicant exposures. After stability, the response criterion was inverted so IRTs had to be *longer* than the longest 75% of the previous 10 IRTs. This established a low response rate. The question was whether methylmercury altered the rate of transition and, if so, how. Exposed rats took a longer time to acquire this low-rate percentile schedule than controls in a dose-related fashion. The change in responding was driven by changes in long IRTs and bout length. No change was seen in the duration of very short IRTs (i.e., those that occur within a bout). The transition to a low-rate schedule shortened bout length in all groups but more slowly in exposed animals. It also increased the number of long IRTs but had little impact on the range of these IRTs. Thus, a response requirement of waiting between responses was met not by changing short within-bout IRTs, or the duration of long interbout intervals, but rather by breaking the bouts into shorter components with more long pauses. It is important to note that this provides a behavioral mechanism for the construct of response inhibition, an important descriptor of many pharmacological or other neurobiological interventions that damage the prefrontal cortex (Mostofsky & Simmonds, 2008). That mechanism is the selection or reinforcement of preestablished long IRTs.

The partitioning of responding into response bouts reveals mechanisms by which behavior changes during transition states in a way that is not possible by examining response rates alone. Interventions that specifically target the value of reinforcers, like extinction and pre-feeding, influence behavior during transition states by their actions on the bout-initiation rate. Interventions that target the pattern of responding, like adding a tandem FR schedule to a VI or reinforcing long IRTs, do so by changing the bout length. A further conclusion is that NCR acts differently than extinction and pre-feeding by altering the bout length but not bout-initiation rate or within-bout rate. Certain transition states are also mediated by bout lengths and long IRTs. In contrast, within-bout rates are relatively difficult to change with the interventions described in this section.

## Bout Identification and Measurement

**Fig. 1:**
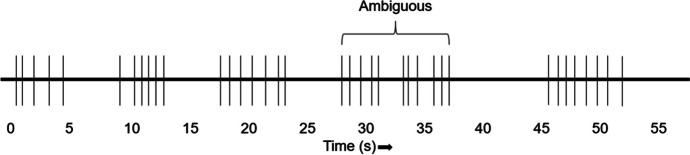
A Difficulty of Bout Measurement. *Note*. Schematic adapted from Shull et al. (2001)

To examine behavior in the two-mode framework, response bouts and interbout pausing, one must measure the rate of bout initiations, the number of responses per bout, and the rate of responding within a bout. This poses a considerable challenge, because it is generally difficult to objectively know and precisely define what constitutes a bout (Shull, 2011). Often the clinician or researcher must analyze a string of discrete responses (e.g., instances of self-injurious behavior [SIB] or runs of lever presses) and examine their general patterning to partition the responses into bouts. This was accomplished by using somewhat subjective criteria before Shull et al.’s (2001) article. Consider the illustration in Fig. 1. Here, responding appears to occur in bouts and would be consistent with the two-mode view of behavior. However, this example also illustrates some of the difficulties with discerning bout features. How many bouts of behavior occur between the 25-s mark and the 40-s mark? It is ambiguous, as one could reasonably argue there are one, two, or three bouts here depending on how the time intervals between responses are viewed. Is the slightly longer IRT after the fifth response simply a longer within-visit pause, or should it be viewed as an unusually short between-visit pause^4^ (Palya, 1992; Shull, 2011; Shull et al., 2001)?

Thus, bouts are easy to describe conceptually but, prior to Shull et al. (2001), they were difficult to measure with confidence. Researchers previously attempted to resolve this ambiguity using a variety of objective methods to partition discrete instances of behavior into bouts. One seemingly straightforward approach is to specify a cutoff for an IRT (e.g., 5 s) and record each response with an IRT < 5 s as constituting a within-bout response (Mellgren & Elsmore, 1991). This tactic was often used in basic laboratories and in applied settings by practitioners because it is seemingly straightforward; however, it is often unclear when one bout of responses ends and another begins. Given that the frequency distributions of within-bout responses and bout initiations often overlap, this method is prone to misclassification of some within-bout responses as bout initiations (or vice versa; Berdoy, 1993; Machlis, 1977; Sibly et al., 1990; Slater & Lester, 1982; Shull et al., 2001; Tolkamp & Kyriazakis, 1999). In addition, as the IRT cutoff is varied, the number of bout initiations and number of within-bout responses must be negatively correlated; this prevents these two response characteristics from being assessed independent of one another and it makes the conclusions dependent on the decision about where the cutoff lies (Shull et al., 2001). Although there is an objective component to this method, it is still ultimately an arbitrary approach for measuring bouts: the cutoff selected to define a bout is bound to misclassify a sizeable proportion of responses and ultimately affect the obtained number and durations of bouts (Shull, 2011). Moreover, comparing results across studies is difficult when investigators use different IRT cutoffs.

Shull et al.’s (2001) approach to partitioning responses into bouts overcomes the criticisms of the IRT-cutoff approach. Shull’s technique is somewhat radical in that it does not attempt to classify individual responses as either representing bout-initiations or within-bout responses based on a priori definitions, cutoffs, of what defines a bout (e.g., any response with an IRT < 5 s). Rather, this approach estimates these quantities (viz., bout initiations and within-bout responses) based on characteristics of the frequency distributions of obtained IRTs. This approach is predicated on the idea that there are two functionally distinct frequency distributions of IRTs, one for pauses between bouts and one for response patterns occurring within a bout, that can be assayed using visual and mathematical techniques described below. Unlike the IRT-cutoff approach, Shull’s method acknowledges the reality that the two distributions likely overlap so a specific IRT may come from one distribution or the other but with different probabilities.

## Log Survivor Plots and Parameter Estimation

**Fig. 2:**
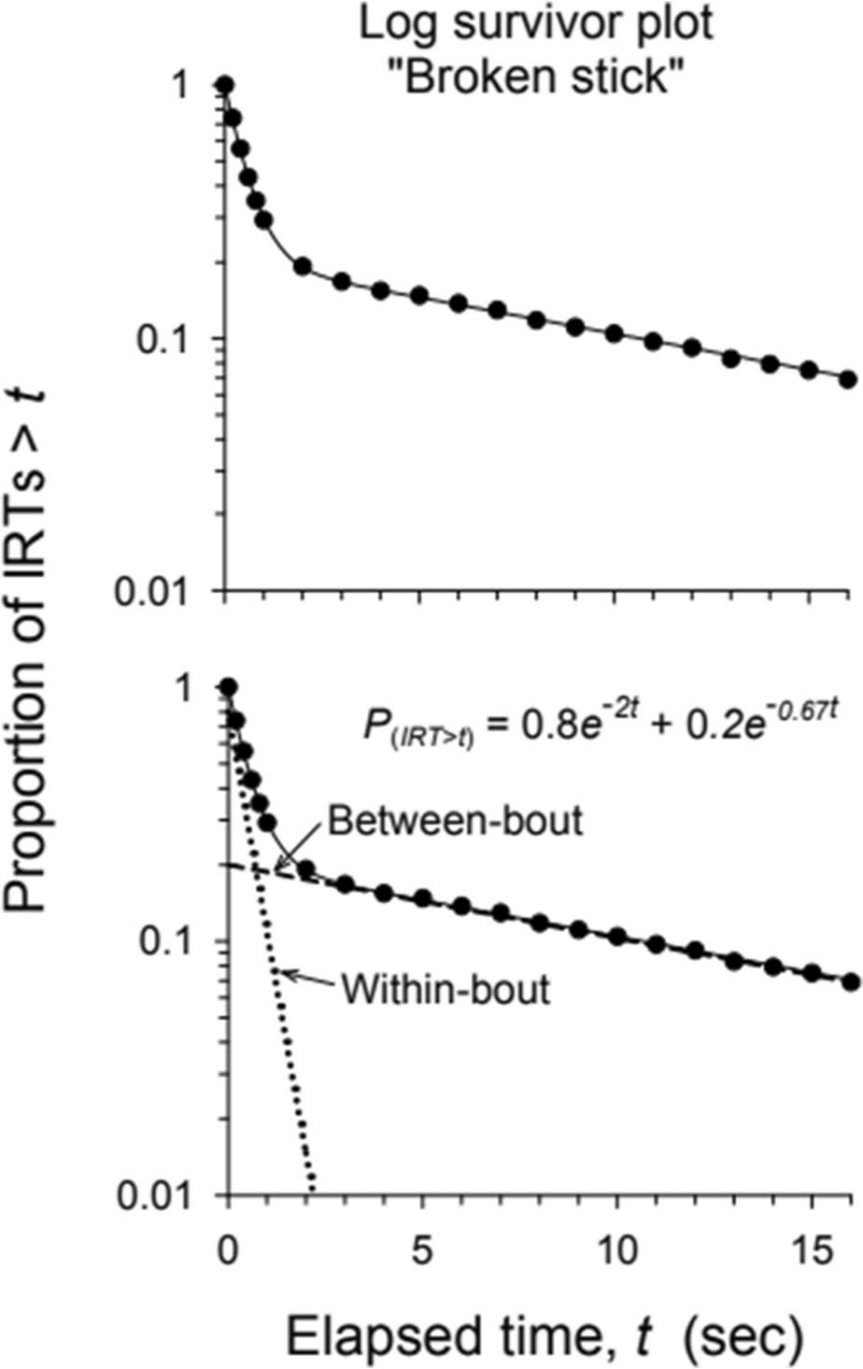
“Broken Stick” Pattern in Log Survivor Plot. *Note*. Figure reproduced with permission from Shull (2011)

Log survival analyses of operant behavior typically reveal a mixture of two exponential distributions—one corresponding to IRTs within ongoing response bouts and the other corresponding to intervals between bouts (e.g., Brackney et al., 2011; Hoffman, 2015, cf. Tanno, 2016). Examination of such a plot (Fig. 2) reveals this two-mode character of responding (Shull et al., 2001).

A log survivor plot shows the proportion of IRTs that are longer than some time *t*. Thus, it shows the proportion of IRTs (*y*- axis) that survive *t* seconds (*x*-axis). It is usually plotted on a semi-logarithmic scale. The IRTs are taken from all, or maybe a representative sample, of unconstrained IRTS that are longer in duration than some time (*t*_*i*_). The *y-*axis is typically logarithmic. In Fig. 2, the first data point says that all the IRTs (proportion = 1.0) are longer than the shortest one and approximately 0.1 of the IRTs are longer than about 12–13 s. Log transforming the *y*-axis allows a large span of IRTs to be visualized and it linearizes the functions.

Two-mode responding is indicated by a characteristic “broken stick” or “hockey stick” appearance indicating high-rate responding within bouts and low-rate responding composed of between-bout IRTs (Kulubekova & McDowell, 2008; Shull et al., 2001, 2004). This broken stick appearance consists of two distinct limbs: the steeply sloped left limb of the log survivor plot corresponds to short within-bout IRTs and the more gradually sloped right limb corresponds to long between-bout pauses (see illustrative example in Fig. 2.).

This two-mode conceptualization of responding can be modeled as the sum of two Poisson processes that give rise to exponentially decaying curves describing the waiting times between two events (Killeen et al., 2002; Shull et al., 2001). Shull and Grimes (2003) provided the following double exponential equation to describe the log survivor plots:

1 $$r\left(t\right)=\left(1-p\right) {e}^{-wt}+p {e}^{-bt}$$

in which r(t) is the proportion of IRTs longer than a given amount of time (t), *p* is the proportion of responses that are bout initiations, *w* is the estimated within-bout response rate, *b* is the estimated rate of bout initiations, *t* represents time since the last response (usually in seconds) and *e* is the base of the natural logarithms (Table 1). Thus, the first term to the right of the equality sign, $\left(1-p\right) {e}^{-wt}$, corresponds to the portion of the log survivor plot associated with within-bout responses. The second term, $p {e}^{-bt}$, corresponds to the portion of the log survivor plot associated with between-bout responses. Here, *p* represents the proportion of IRTs that are bout initiations, and *b* is the rate of initiating bouts (Table 1). If you draw a straight line through the right limb of the “broken stick” plot back to the y-axis, its *y*-intercept line is *p*. The reciprocal of *p* (i.e., 1/*p*) plus 1 provides an estimate of the average number of responses per bout (i.e., bout length). Adding 1 is required because the unit of analysis is the IRT and it takes two responses to produce one IRT, three responses to produce two IRTs, and so forth. For example, a *p* value of 0.2 would correspond to a mean bout length of six responses. In visual terms, the lower the *y*-intercept of the right limb, the longer the average bout is (Shull et al., 2004). In conceptual terms, the more responses in the bouts, the smaller the proportion of responses that initiate bouts. The equation describing this function is shown in the bottom panel of Fig. 2. In this example, the within-bout rate is 2 responses/s, the slope of the faster-descending line. The bout-initiation rate is 0.67 bouts/s. The value of *p* is 0.2 so the average bout length is 1 + 1/*p* = 6.

**Table 1:** Bout Elements

Parameter (Eq. 1)	Interpretation	Detail	Hypothetical Parameter Value	Example	Interpretation (Reciprocal)	Example (Reciprocal)
*b*	Bout-initiation rate	A bout initiation is the first response in a set of responses for a given bout	0.05	.05 resp/s (3 bouts initiated every min)	Pause duration in-between bouts	1/.05 = 20-s pause in-between bouts
*p*	Proportion of responses that are bout-initiations	Corresponds to the probability of quitting a bout	0.25	One-quarter of all responses are bout initiations	Number of IRTs within a bout	1 + 1/.25 = 5 barks*
*w*	Within-bout response rate	Within-bout responses correspond to responses occurring in a given bout once the bout has been initiated	0.2	.2 resp/s (barks occur at a rate of 12 barks per min)	Average within-bout IRT	1/.2 = one bark every 5 s

^*^Adding 1 to the number of IRTs [(1/p) + 1] corresponds to the number of total responses constituting a given bout including the bout-initiation response

Three important measurement considerations are easy to overlook when modeling using log survival analysis. One is the estimate of bout length, made by adding 1 to 1/p. Failing to add 1 here would omit the bout-initiation response. Second, the model in Fig. 2 assumes that the shortest IRT is 0 s, but that is physically impossible. There is always a non-zero minimum IRT just as there is a maximum response rate. A minimum IRT exists because a response must have some duration and there is always a minimum duration. An IRT is typically measured from the onset of one response to the onset of a second response. A “true IRT” (Killeen & Sitomer, 2003) is measured from the *offset* of one response to the onset of another, removing response duration from the measure, but that is usually not measured. An IRT cannot be initiated or terminated while engaged in the response; therefore, the shortest response duration represents the shortest IRT possible in a particular situation. This is termed the refractory period. As described above, the refractory period reflects limits on responses rates imposed by biophysical constraints such as the device measuring the response (lever, running wheel) and the anatomy and physiology of behavior (limb length, muscle strength; Brackney et al., 2011; Killeen, 2023; Killeen et al., 2002).

Failing to consider the refractory period can significantly distort the estimates of the measures of microstructure. Two approaches have been taken to address this issue. One is to add a term to the regression equation used to estimate the bout parameters to represent this refractory period (Brackney et al., 2011). This refractory bi-exponential equation adds to the complexity of the model used to estimate bout parameters but can enhance the fit significantly, as determined by an improvement in the AICc. It does appear to introduce a small bias in the estimate of the smallest IRT, possibly because of an assumption that the IRTs are normally distributed. A second approach is to let the minimum IRT in the obtained distribution serve as the refractory period (Shen et al., 2016a, 2016b). Here, the minimum IRT is subtracted from all IRTs so the shortest one in the regression equation is 0 s, allowing the log survival function to be fit accurately.

**Fig. 3:**
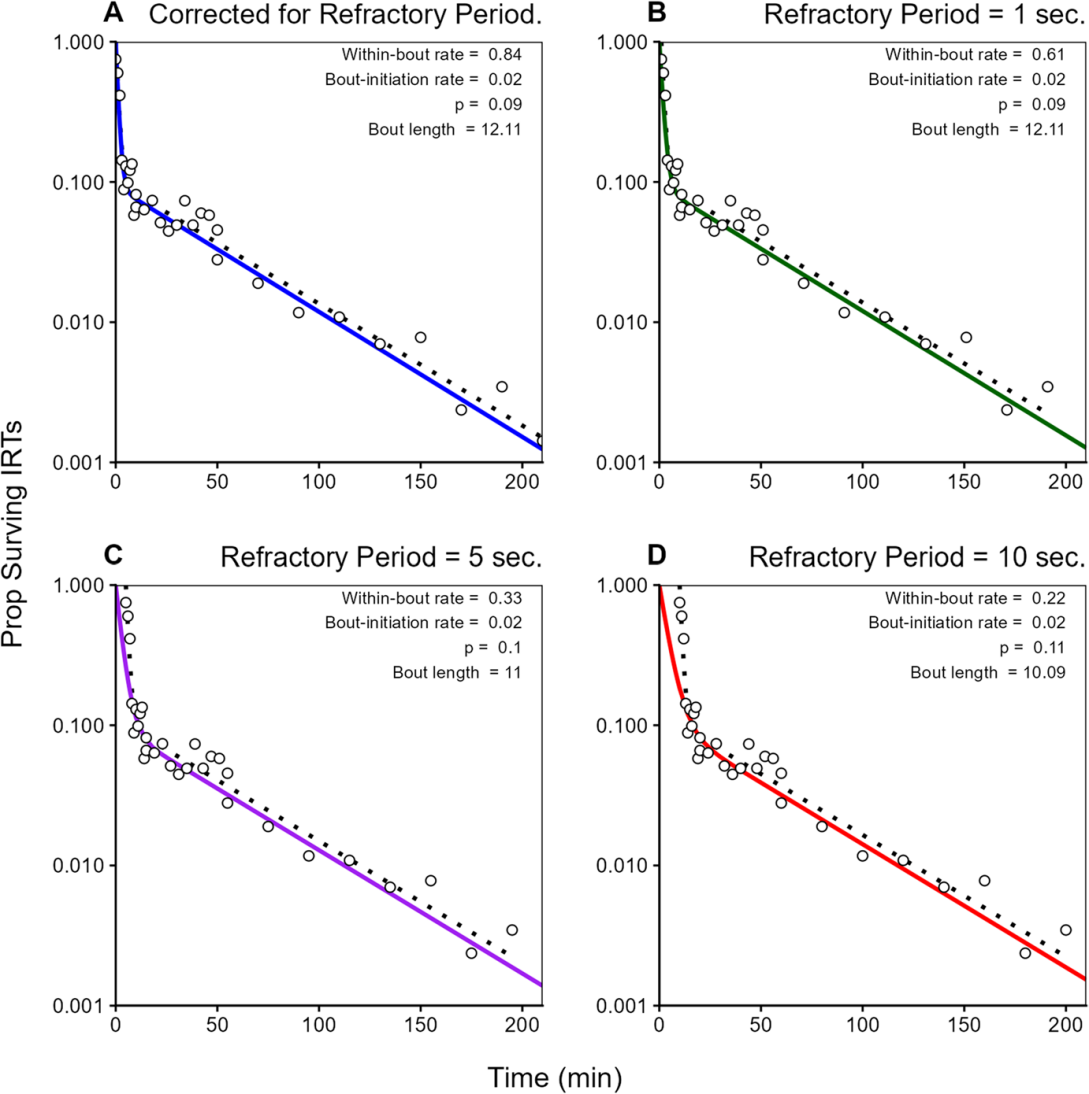
How the Refractory Period Distorts Estimates of Bout Parameters. *The* circles and dotted black lines show the simulated survival plot. The top left panel shows a bout analysis after subtracting the minimum IRT. The other panels show data that would be obtained if the refractory period is not subtracted. The solid, colored lines show the best fit to the uncorrected data

Figure 3 illustrates how a failure to consider the refractory period distorts the estimation of the bout parameters. A log-survival curve was generated using the parameters of *w* = 0.8, *b* = 0.02, and *p* = 0.8 and then random noise was added. The dotted black line and circles in Fig. 3A draw, respectively, the function and the function plus noise. Panel A shows the best fit to these data (solid blue line), with the estimated coefficients shown in the top right portion of the panel. The fitted curve differs slightly from the generating function because of the random noise. This curve would result after subtracting the refractory period from each of the other panels, so it is called the “corrected” curve. Panel B illustrates a refractory period of only 1 s, or 0.5% of the longest IRT of 200 s. There is no discernable impact on the appearance of the curve, but even this small refractory period reduced the estimated within-bout response rate from 0.84 to 0.61 responses/s. The longer refractory periods of 5 s (Panel C, solid purple line) and 10 s (Panel D, thick red curve) show even more distortion.

The 10-s refractory illustrates the distortion dramatically. Because the refractory period is 10 s, the entire function is shifted to the right by that amount. This would arise if the shortest response duration was 10 s, as might be seen if the response was, say, working math problems or folding towels. Fitting a log-survival plot to the uncorrected data produces an erroneous estimate of the within-bout rate and the bout length. The reason is clear in this panel: the log-survival equation forces the best-fit line to intercept the y-axis at a value of 1.0, pulling the left curve downward and to the left, forming a shallower slope and smaller value for *w*. Subtracting the shortest IRT corrects for this, as would including a term in the Eq. 1 for this refractory period.

A third consideration when fitting the function will only be mentioned without illustration. A typical log-survival curve spans two or more orders of magnitude. The expected variance around the larger proportions (left side of the curve) will likely be larger than for the smaller values, and this can distort the regression analysis. Reciprocal-Y weighting has been recommended to correct this distortion (Kessel & Lucke, 2008; Shull & Grimes, 2003). The reciprocal-Y weighting rule assigns weights to data points that are inversely proportional to their values. This means that data points with larger values are weighted less than data points with smaller values, which prevents very long IRTs from disproportionately influencing the fits of the curve. Most nonlinear regression packages have a term for weighting so this entering 1/Y (or 1/Proportion of IRTs > *t*) here would accomplish this task. We have sometimes found that reciprocal Y^2^ weighting is warranted. The best way to determine whether weighting is necessary is to try the fit with and without the weighting function and examine the fit against the obtained data or, better, inspect the residuals for the absence of a pattern.

## Operations Affecting Bout Structure

**Fig. 4:**
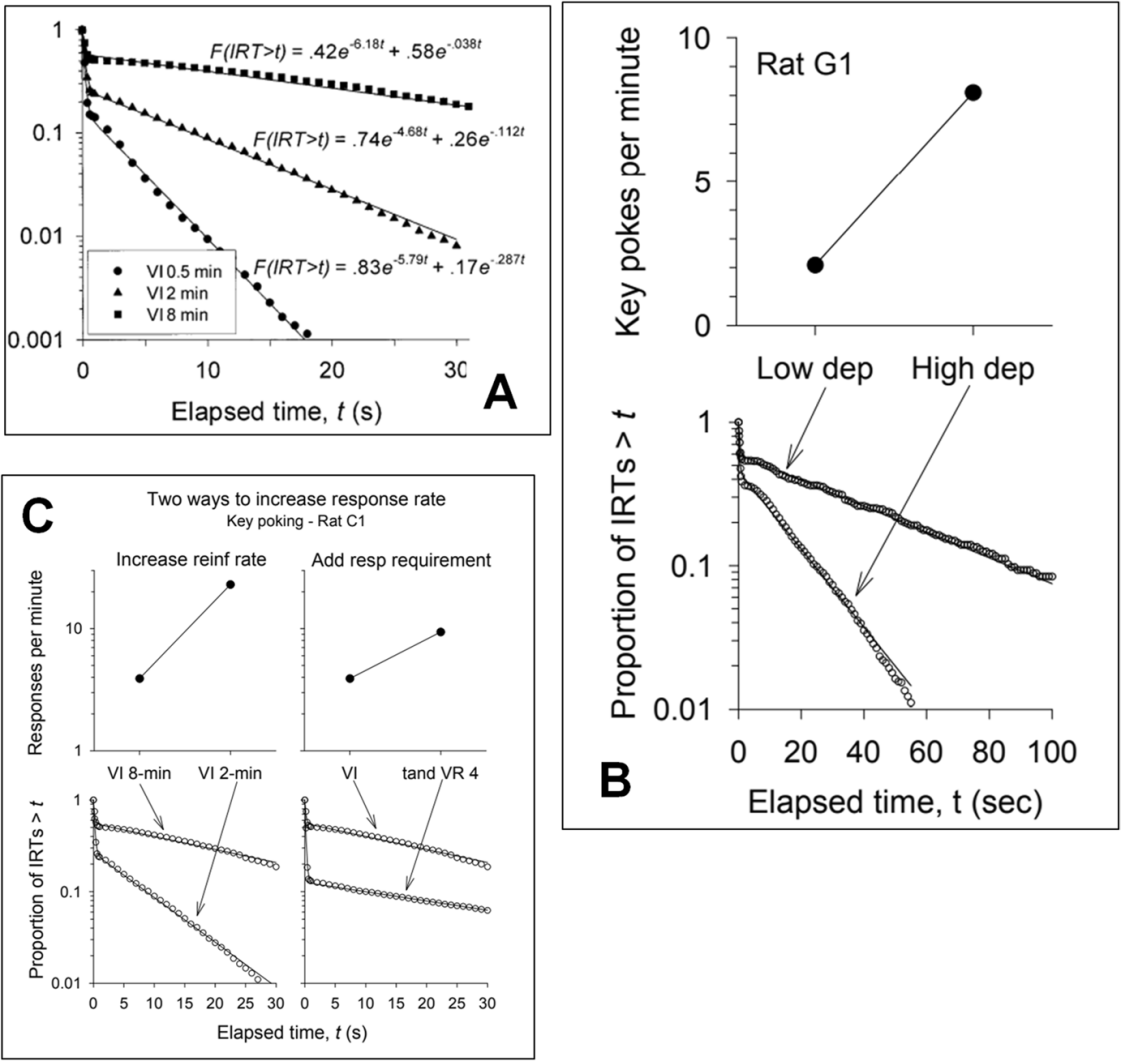
Illustrative Log Survivor Plots and Fits of Eq. 1. *Note*. Figures reproduced with permission from Shull et al., (2004; right panel) and Shull (2011; top left and bottom left)

As described above, a number of operations involving schedule manipulations (e.g., rate or magnitude of reinforcement, punishment contingencies, addition of alternative reinforcement; Johnson et al., 2009) pharmacological manipulations (e.g., exposure to specific drugs or toxicants with known motor effects; Newland et al., 2015), and effort-related manipulations (e.g., response force or duration requirements; Brackney et al., 2021) have all shown to affect at least one of these components selectively. Here, we begin to introduce the quantitative measures of response bouts as described by Shull et al. (2004). As depicted in Fig. 4A, increasing the reinforcer rate by making the VI schedule richer does not affect the steep slope of the left (within-bout) limb, but it does steepen the slope of the right (between-bout) limb (Shull et al., 2004). This steepening of the right limb indicates a shift of the frequency of between-bout intervals towards shorter durations. In other words, the duration of pauses between bouts is diminished (or the bout-initiation rate increases) whereas within-bout response rate remains unchanged (Shull et al., 2004). Increasing the reinforcer rate also lengthened the left limb, implying that relatively more of the IRTs emitted within the session consist of within-bout IRTs. This means that the average number of responses per bout also increased as a function of the higher reinforcer rate (Shull et al., 2004). Note that the intercept of the right limb decreases from 0.58 (2.7 responses/bout) to 0.26 (4.8 responses/bout) as the reinforcer rate increases. In short, as the reinforcer rate increased, the rats initiated bouts of responding more quickly and the bouts lengthened, but the rate of responding once a bout commenced was unchanged. This result illustrates the important distinction between the length of the limbs and their slopes. Similar effects are observed following manipulation of a different motivational factor, namely providing additional food (Fig. 4B). In the top panel of Fig. 4B it is seen that increasing food deprivation (“High Dep”) increased response rate. In the bottom panel it is seen that the rate increase occurred because of a higher bout-initiation rate. Bout length also increased.

The effects of a change of reinforcement schedule or reinforcer rate are shown in Fig. 4C. The left two panels show that increasing reinforcer rate, a motivational variable, increased response rate by increasing the bout-initiation rate (steeper slope of the right limb). The contrasting effect of imposing a tandem VI FR schedule is shown in the right two panels. Here, a rat’s responding was maintained by a VI schedule of reinforcement with exactly one (VI) or an average of four (tandem VI VR 4) responses required after the VI timed out. The VR schedule increases response rates and selects short IRTs (Rasmussen et al., 2022; Shull et al., 2001; Zeiler, 1977). This primarily affected the left limb of the log-survivor plots while leaving the slopes of the right limb unchanged (Fig. 4C, bottom panel). Such a manipulation might be viewed as imposing a speed requirement, if indirectly, for responding but without changing motivation. For example, if a client is required to fold towels or complete worksheets, this could model requiring that such tasks be completed quickly or saying that, after the task is eligible for reinforcement, at least four more must be completed.

## Failures to Replicate Two-Mode Responding

There is substantial experimental evidence supporting the idea that operant behavior is organized into a series of bouts of engagement separated by periods of disengagement (Shull, 2011), but exceptions have been noted. Much of the work investigating two-mode responding via log survivor plots has been performed with nonhuman animals (primarily rodents), but this work has been extended to schedule-controlled behavior in several laboratory human-operant preparations (Chen & Reed, 2020, 2021, 2023; Chen et al., 2020; Reed, 2011, 2015, 2020; Reed et al., 2018).

Attempts to generalize the results of log survivor analyses to pigeons have been both unsuccessful and informative (Bennett et al., 2007; Bowers et al., 2008; Davison, 2004; Podlesnik et al., 2006; Smith et al., 2014; see also Kulubekova & McDowell, 2008). The failures led to a deeper understanding of when two-mode responding arises. Pigeons’ log survivor plots do not typically show the broken-stick pattern indicative of two-mode responding organized into bouts and pauses. Rather, plots for pigeons are usually single limbed and approximately linear on semi-log axes (Smith et al., 2014), indicative of a single mode. In visual terms, this produces log survivor plots without an identifiable change in slope. This means that pigeons’ responding is not organized into bouts at the reinforcer rates and under the conditions commonly used in pigeon studies but is better described by a single exponential distribution than the double exponential model described by Eq. 1. This decreasing straight line means that responding approximates that generated from a random emitter—the steeper the line, the higher the emission rate (Shull et al., 2001). It implies that the pigeon was never disengaged or that the theory does not hold for pigeons.

Many hypotheses have been advanced to explain the curious but reliable finding that pigeons’ key pecking does not produce a broken stick log survivor function under typical schedule arrangements. In an attempt to identify the conditions necessary for single versus two-state responding, Shull (2005) reanalyzed 18 datasets from a number of rat and pigeon studies using Herrnstein’s (1970) hyperbolic model. He found that estimates for *R*_*e*_ (alternative background reinforcement, sometimes called *R*_*o*_ for other reinforcers) were generally lower in pigeon studies than in rat studies. This is significant because an organism that obtains little reinforcement from alternative sources will be less likely to sample from those sources for an extended period of time vis-à-vis extended periods of between-bout responding (Bennett et al., 2007). In other words, different log survivor functions may occur for pigeons compared to rats because pigeons obtain less reinforcement in the operant chamber from nonprogrammed sources (e.g., grooming, chewing, drinking; Smith et al., 2014). It follows from this hypothesis that responding should more closely follow the two-mode pattern when alternative background reinforcement is increased or when the rate of reinforcement for the target response is decreased (e.g., Johnson et al., 2009; Smith et al., 2014).

**Fig. 5:**
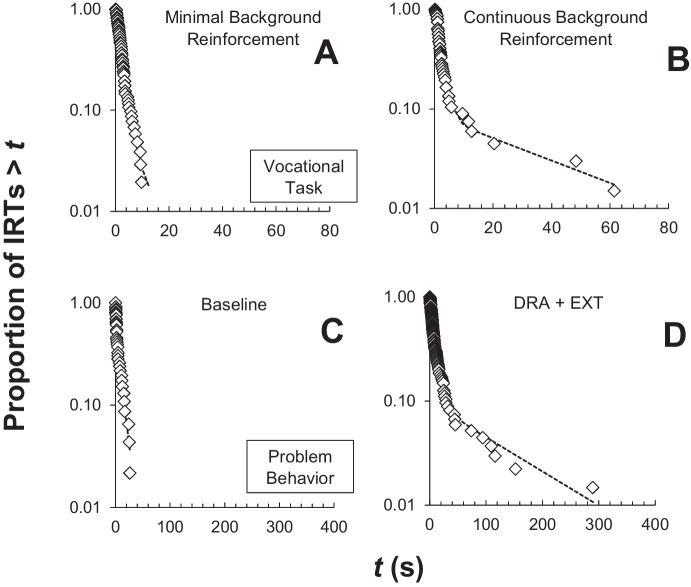
Effects of Alternative Reinforcement on Bout Organization

The absence of a broken stick log survivor function could be diagnostic of a barren environment or a limited repertoire for extracting background reinforcement from the environment. To illustrate, consider the following example with real-world data obtained from an individual (“Jonah”) who was admitted to a hospital-based inpatient unit for the assessment and treatment of severe behavior. During one assessment from a prospective research study evaluating the effects of background reinforcement on task completion, Jonah earned tokens (which were exchanged for a small amount of food after each 5-min session) on a VI-60 s schedule for working on a single-operant task (e.g., placing small items into a container). There were no consequences for challenging behavior, and the data collector did not interact with Jonah during the session apart from delivering tokens. Log survivor plots generated from these sessions did not consistently evidence the broken-stick appearance indicative of responding organized into bouts (Fig. 5A). This could be attributable to the minimal degree of background reinforcement available for discontinuing the visit to the target response. When the therapist increased background reinforcement by providing continuous verbal attention, which was a functional reinforcer for his challenging behavior (Iwata et al., 1982/1994), the log survivor plots changed to evidence a clear two-mode pattern of responding (Fig. 5B). These outcomes mirror findings from Smith et al. (2014), who reported a similar effect with pigeons, but only when a changeover delay (COD) was present to create a consequence for changing the response and reinforcer. Such findings are consistent with Shull’s notion that the appearance of a broken-stick pattern in the log survival analysis is influenced by the presence of alternative reinforcers. It might be worth noting an important distinction between the illustrative data from Jonah and the results from Smith et al. (2014). In Smith et al.’s study, the pigeon could only perform one task at a time. In Jonah’s situation, it was possible to perform the task and converse simultaneously (i.e., a concurrent schedule). As has been noted previously, there is much more to understand about the role of background reinforcers on operant performance (Soto et al., 2005).

Consider illustrative data from a treatment evaluation for attention-maintained challenging behavior for one patient (“Nick”; see Falligant et al., 2021). Log survivor plots were generated from IRTs obtained from his final baseline session and subsequent treatment sessions (which consisted of differential reinforcement of a functional communicative response combined with extinction). Similar to Jonah, responding did not evidence the broken-stick appearance during baseline (Fig. 5C). It only became organized into bouts following the introduction of alternative reinforcement and extinction for challenging behavior (Fig. 5D).

Another issue sometimes found with pigeons that is hypothesized to lead to deviations from two-mode responding is the presence of multiple inflection points attributable to recurrent pecking or “nibble pecks” (Bowers et al., 2008; Killeen et al., 2002; Palya, 1992; see Pinkston & Moore, 2020, for a similar finding with rats). This could introduce a third leg to the left end of the curve because the nibble pecks occur at a very high rate. Yet another hypothesis is that, compared to rodents, nontarget behaviors and activities that occur for pigeons during periods of disengagement are considerably more variable and thus contribute to the lack of a consistent delineation of IRT distributions characterizing bouts and pauses (Davison, 2004). Thus, differences in bout structure may reflect limited background reinforcement in the environment, the availability of activities that take differing lengths of time to engage, behavioral variability, and/or generalized response tendencies corresponding to distinct behavioral phenotypes across species and organisms (e.g., BALB/c vs. C57BL/6 mice; see Fig. 3 in Johnson et al., 2009). Such activities could introduce additional legs to the log survival plot. In our experience, sometimes a single third leg has been seen on the right end of the function, indicative of three states of responding. This might be interpreted as bouts of bouts of responding. To give a simple example, an animal might switch between exploring the operant chamber and lever pressing, as described above. If, during long sessions, it took brief, episodic naps, then the two-state model (exploring and lever pressing) would alternate with naps and the latter would appear as very long IRTs.

## Directions for Applied Clinical Research and Practice

This bout-analytic framework can yield valuable information regarding the underlying temporal dynamics of behavior by (1) facilitating analysis of the dissociable contributions of motoric and motivational variables on operant performance and (2) providing a quantitative assay of the impact (or lack thereof) of interventions such as alternative reinforcement, motivational operations, reinforcement schedules, response difficulty, or cost, among others (see Marr, 1992). This approach lends itself to the analysis and individualization of disruption tactics for challenging behavior involving a variety of procedures. For example, Brackney et al. (2017) found that extinction and pre-feeding (motivational manipulations) decrease responding by selectively reducing the rate of bout initiations. In contrast, NCR decreases responding by selectively reducing the length of bouts. The effects of these and other procedures—including response blocking, the application of protective equipment, or tactics directly manipulating response effort—on the bout structure of challenging behavior should be explored in applied research.

**Fig. 6:**
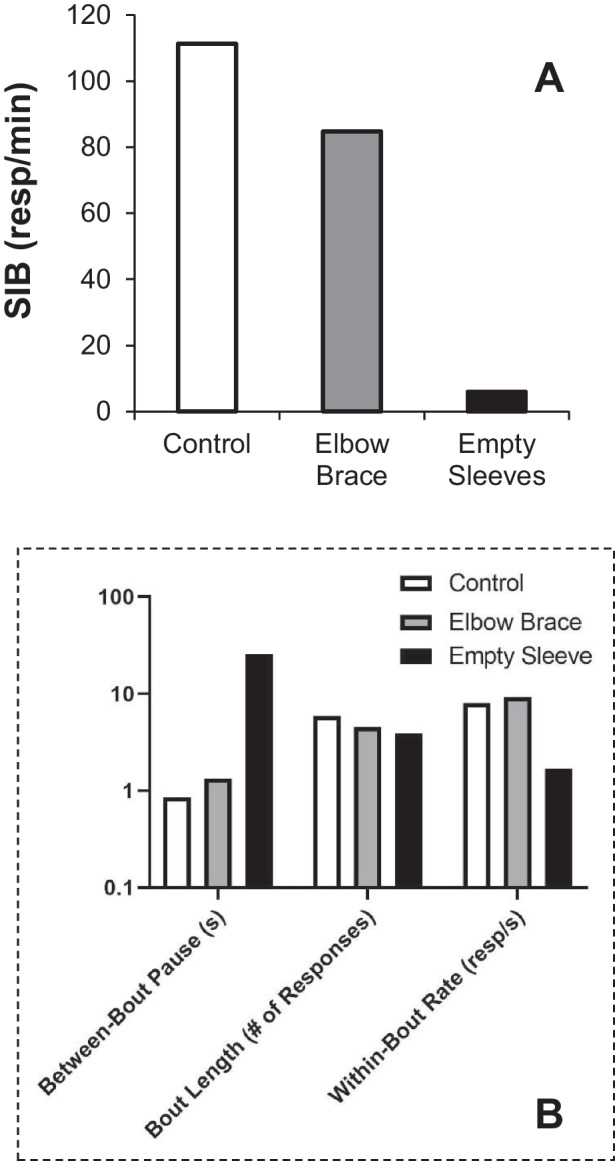
Isolating the Disruptive Effects of Protective Equipment on Self-Injurious Behavior. *Note*. Sessions were terminated early during control and elbow brace conditions, thus high rates of behavior do not reflect the obtained frequency of SIB but rather the rate during the sessions prior to termination. Note log scale on vertical axis in panels B

To illustrate how increasing response effort affects within-bout rate and bout-initiation rate with a clinical example, consider data from an assessment evaluating the effects of protective equipment on SIB (i.e., self-biting, head banging, hand-to-head SIB) for an individual from an inpatient unit (Participant #1 from Rooker et al., 2022) in Fig. 6. Relative to a no-equipment control condition, one item (empty sleeves) greatly decreased SIB whereas another form of protective equipment (elbow brace) did not (Fig. 6A). Log survivor plots from the control and elbow brace conditions indicated considerable overlap between the distributions corresponding to the within-bout IRTs and between-bout IRTs. Nonetheless, two limbs could be isolated quantitatively using Eq. 1 (Fig. 6B). Interpretation of parameter estimates obtained with Eq. 1 indicate the empty sleeves worked primarily by increasing the duration of pauses in-between bouts of SIB, equivalent to decreasing the bout-initiation rate, and offering evidence of decreased motivation to self-injure. At the same time, the sleeves decreased the within-bout response rate, suggesting that the sleeves made the self-injuring behavior more difficult to execute. It is interesting that bout length remained unchanged. This resembles the aforementioned findings from Brackney et al. (2011) in which making a response motorically difficult can also decrease the motivation to execute it.

Bout analyses may be useful for understanding automatically maintained self-injurious behavior (ASIB). In the majority of cases, SIB is maintained by social-positive (e.g., caregiver attention) or social-negative (e.g., termination of instructional demands; Melanson & Fahmie, 2023) reinforcement. However, in some cases SIB persists in the absence of socially mediated consequences and is said to be automatically maintained (Vaughan & Michael, 1982). Within this class of automatically maintained behavior, there is considerable heterogeneity in the environmental conditions under which SIB is observed and in its sensitivity to disruption by alternative reinforcement (see Hagopian et al., 2015, 2017, 2023). Subtype 1 SIB is characterized by a high level of differentiation (LOD) between the no-interaction and toy-play control within a functional analysis (FA) and is very responsive to reinforcement-based interventions. In contrast, Subtype 2 SIB is characterized by a low LOD and is resistant to reinforcement-based interventions (Hagopian et al., 2018). One hypothesis for this distinct response dynamic between subtypes relates to the putative endogenous consequences of SIB. The relative insensitivity of Subtype 2 SIB to disruption by alternative reinforcement suggests that it produces higher magnitude endogenous reinforcement relative to Subtype 1 SIB. Although this hypothesis is difficult to test directly, this bout-analytic framework could elucidate potential differences in operant performance attributable to differences in reinforcement magnitude or quality pertinent to ASIB.

**Fig. 7:**
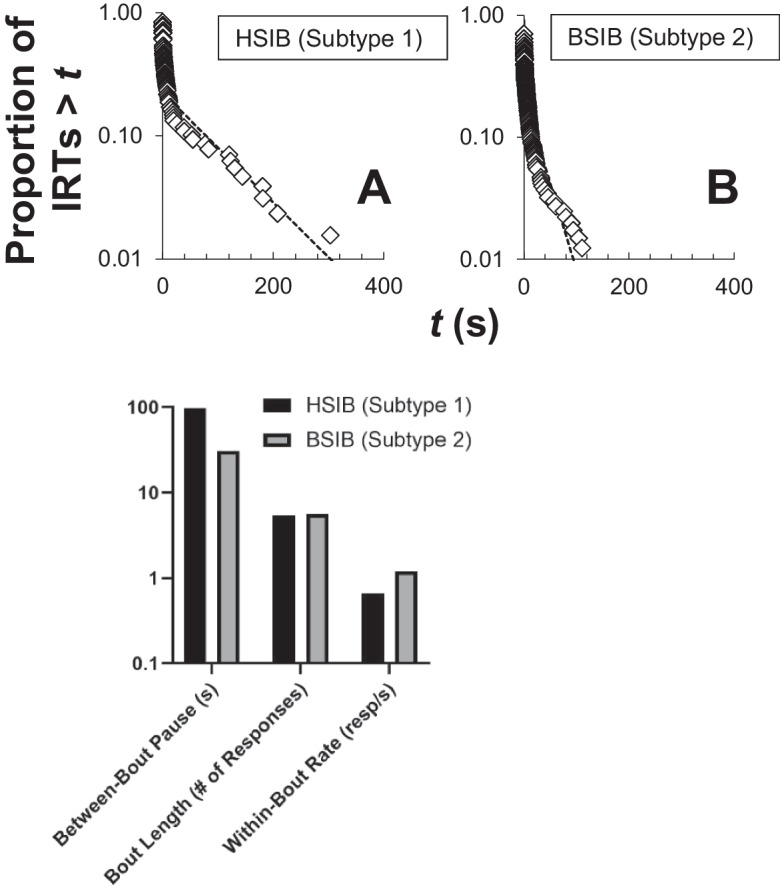
Examining Differences in ASIB Subtypes. HSIB = head-directed self-injurious behavior; BSIB = body-directed self-injurious behavior. Note log scaling on all vertical axes for log survivor plots (panels A-B) and parameter interpretation (panel C)

To illustrate how this might be explored, consider FA data from “Jack,” a young boy receiving intensive behavioral services on the same inpatient unit described in the previous examples. FA results indicated that Jack’s head-directed SIB (HSIB) met criteria for Subtype 1 whereas body-directed SIB (BSIB) met criteria for Subtype 2. We constructed log survivor plots for HSIB and BSIB IRTs from the relevant alone FA sessions used to subtype these topographies and fit Eq. 1 to these data (Fig. 7). The log survivor functions for HSIB (Fig. 7, top left) and BSIB (Fig. 7, top right) both evidenced the broken-stick pattern, indicating that responding was organized in bouts. It should be noted that for BSIB the left limb of this function was considerably longer, and the slope of the right limb was much steeper compared to HSIB. Fits of Eq. 1 reveal that the estimated bout length for HSIB (5.5 responses) and BSIB (5.6 responses) were nearly identical (Fig. 7, bottom panel). Estimates of within-bout response rate were different for HSIB (0.7 resp/s) and BSIB (1.2 resp/s)—the slightly higher rate estimate for BSIB could indicate this response is less effortful than HSIB. The biggest difference in these topographies of SIB appeared to be the much shorter pause duration in-between bouts of Subtype 2-BSIB (30.6 s) relative to Subtype-1 HSIB (97.5 s). Recall that differences in bout-initiation rate are attributable to motivational variables, so this example could be viewed as supporting the hypothesis that differences in the magnitude of reinforcement of SIB (at least for this case) may be a distinguishing factor for Subtype 1 and 2 SIB. In a recent study, Falligant (2024) found that, in a retrospective analysis of clinical cases (*N* = 20), nearly all log survivor plots corresponding to Subtype 1 SIB were characterized by a clear “broken stick” appearance. In contrast, the log survivor plots corresponding to individuals with Subtype 2 SIB generally had no such appearance or only faint evidence (as in Fig. 7). These plots tended to consist of only a single limb evidencing a single, extremely steep exponential decay function, similar to the plots produced by pigeons described above. SIB classified as Subtype 1 is much more likely to evidence this “broken stick” pattern, indicative of responding organized in bouts, relative to Subtype 2 SIB. For the latter, the distribution of IRTs appeared to consist of a single exponential function that occurs for behavior without a coherent temporal organization (similar to findings from log survivor plots obtained with pigeons; e.g., Podlesnik et al., 2006). This raises the hypothesis that there is no credible alternative reinforcer for Subtype 2, as compared with Subtype 1, and/or that subtypes fundamentally differ in terms of the temporal organization of their SIB. As described above, this is potentially significant because the temporal organization of responding underlies behavioral momentum and persistence (Shull et al., 2002) and the relative value of alternative reinforcers. To that end, preliminary results imply it is the relative sensitivity to disruption of bout initiations, specifically by alternative reinforcement, that mediates the differential responsiveness to reinforcement-based interventions between subtypes.

## Conclusions

Operant behavior can be viewed as occurring in bouts and pauses. Bouts refer to periods of engagement in a target response alternating with pauses associated with periods of disengagement from the target response during which the individual engages in other activities. The idea that responding occurs in bouts and pauses has been used in the fields of neurotoxicology and behavioral neuroscience to disentangle the contribution of motivational and motoric variables to the pattern of operant behavior. Analysis of IRTs often reveals a mixture of two exponential distributions. One corresponds to short IRTs within ongoing response bouts, reflecting motor properties of the operant, and the other corresponds to pauses associated with longer intervals between bouts, reflecting the motivation behind the response. Partitioning the ebb and flow of behavior in this manner facilitates analysis of the mechanisms underlying behavior maintenance and change. We hope this primer serves to initiate a new bout of research examining the boundaries of generality of this approach to clinically relevant behavioral phenomena and other areas for translation.

## Data Availability

The data supporting this study's findings are available from the corresponding author upon reasonable request.

## Footnotes

1 As it turns out, analyzing barks in bouts improves the ability to identify individual dogs by their barks rather than analyzing isolated barks (Yin & McCowan, 2004).

2 In a technical sense, this is a second order (RI 30 s [Percentile 10:0.5 S^P^]) schedule (Marr, 1979).

3 These are reinforcement schedules that require completion of two or more successive and unsignaled schedule requirements.

4 This could be attributed to a variety of factors, including an unmeasured response via a data recording error (Palya, 1992) or a subcriterion response (Pinkston & Moore, 2020).
